# Financial Exploitation of Older Adults During the Early Months of the COVID-19 Pandemic

**DOI:** 10.1093/geroni/igab046.333

**Published:** 2021-12-17

**Authors:** Pamela Teaster, Karen Roberto, Jyoti Savla, Chenguang Du, Emily Hoyt, Scott Beach, Neil Charness, Peter Lichtenberg

**Affiliations:** 1 Virginia Tech, BLACKSBURG, West Virginia, United States; 2 Virginia Tech, Blacksburg, Virginia, United States; 3 Center for Gerontology, Virginia Tech, BLACKSBURG, Virginia, United States; 4 University of Pittsburgh, University of Pittsburgh, Pennsylvania, United States; 5 Florida State University, Tallahassee, Florida, United States; 6 Wayne State University, Detroit, Michigan, United States

## Abstract

COVID-19 created a “perfect storm” for financial exploitation directed at older adults. We invited adults aged 60 and older enrolled in gerontology research registries at Virginia Tech, Florida State University, Wayne State University, and University of Pittsburgh to complete an on-line survey about experiences with financial exploitation by strangers. The 997 respondents ranged in age from 60 to 98 (M = 71.3; SD = 6.8); most identified as White (93.4%), female (64.2%) and living with a spouse/partner (58%). Approximately one-half of respondents (56.87%) reported experiencing a scam attempt about COVID-19 issues. Most contact by scammers was electronic (49%) and frequently occurred two or more times (40%). Most respondents ignored the request (i.e., hung up phone, deleted text/email, threw away mail). However, 9% sent the requested payment, and 4% gave the scammer their personal information. Confidence in financial matters and having attended financial educational programs protected older adults from being scammed.

